# Identification of Natural Mutations Responsible for Altered Infection Phenotypes of Salmonella enterica Clinical Isolates by Using Cell Line Infection Screens

**DOI:** 10.1128/AEM.02177-20

**Published:** 2021-01-04

**Authors:** Rafał Kolenda, Michał Burdukiewicz, Marcjanna Wimonć, Adrianna Aleksandrowicz, Aamir Ali, Istvan Szabo, Karsten Tedin, Josefin Bartholdson Scott, Derek Pickard, Peter Schierack

**Affiliations:** a Department of Biochemistry and Molecular Biology, Faculty of Veterinary Medicine, Wrocław University of Environmental and Life Sciences, Wrocław, Poland; b Institute of Biotechnology, Faculty Environment and Natural Sciences, BTU Cottbus-Senftenberg, Senftenberg, Germany; c Warsaw University of Technology, Warsaw, Poland; d National Institute for Biotechnology and Genetic Engineering, Faisalabad, Pakistan; e National Salmonella Reference Laboratory, German Federal Institute for Risk Assessment (BfR), Berlin, Germany; f Free University Berlin, Berlin, Germany; g Cambridge Institute for Therapeutic Immunology & Infectious Disease, University of Cambridge Department of Medicine, Cambridge, United Kingdom; h Faculty of Health Sciences, Public Health Campus, Brandenburg, Germany; Centers for Disease Control and Prevention

**Keywords:** *Salmonella*, adhesion, invasion, infection, clinical isolates, epithelial cells, *sanA*, *dksA*, genomics, transcriptomics, host cell invasion

## Abstract

The initial steps of *Salmonella* pathogenesis involve adhesion to and invasion into host epithelial cells. While well-studied for Salmonella enterica serovar Typhimurium, the factors contributing to this process in other, host-adapted serovars remains unexplored. Here, we screened clinical isolates of serovars Gallinarum, Dublin, Choleraesuis, Typhimurium, and Enteritidis for adhesion to and invasion into intestinal epithelial cell lines of human, porcine, and chicken origins. Thirty isolates with altered infectivity were used for genomic analyses, and 14 genes and novel mutations associated with high or low infectivity were identified. The functions of candidate genes included virulence gene expression regulation and cell wall or membrane synthesis and components. The role of several of these genes in *Salmonella* adhesion to and invasion into cells has not previously been investigated. The genes *dksA* (encoding a stringent response regulator) and *sanA* (encoding a vancomycin high-temperature exclusion protein) were selected for further analyses, and we confirmed their roles in adhesion to and invasion into host cells. Furthermore, transcriptomic analyses were performed for *S*. Enteritidis and *S*. Typhimurium, with two highly infective and two marginally infective isolates for each serovar. Expression profiles for the isolates with altered infection phenotypes revealed the importance of type 3 secretion system expression levels in the determination of an isolate’s infection phenotype. Taken together, these data indicate a new role in cell host infection for genes or gene variants previously not associated with adhesion to and invasion into the epithelial cells.

**IMPORTANCE** *Salmonella* is a foodborne pathogen affecting over 200 million people and resulting in over 200,000 fatal cases per year. Its adhesion to and invasion into intestinal epithelial cells represent one of the first and key steps in the pathogenesis of salmonellosis. Still, around 35 to 40% of bacterial genes have no experimentally validated function, and their contribution to bacterial virulence, including adhesion and invasion, remains largely unknown. Therefore, the significance of this study is in the identification of new genes or gene allelic variants previously not associated with adhesion and invasion. It is well established that blocking adhesion and/or invasion would stop or hamper bacterial infection; therefore, the new findings from this study could be used in future developments of anti-*Salmonella* therapy targeting genes involved in these key processes. Such treatment could be a valuable alternative, as the prevalence of antibiotic-resistant bacteria is increasing very rapidly.

## Abstract (precis)

*Salmonella* is a foodborne pathogen affecting over 200 million people and resulting in over 200,000 fatal cases per year. Its adhesion to and invasion into intestinal epithelial cells represent one of the first and key steps in the pathogenesis of salmonellosis. Still, around 35 to 40% of bacterial genes have no experimentally validated function, and their contribution to bacterial virulence, including adhesion and invasion, remains largely unknown. Therefore, the significance of this study is in the identification of new genes or gene allelic variants previously not associated with adhesion and invasion. It is well established that blocking adhesion and/or invasion would stop or hamper bacterial infection; therefore, the new findings from this study could be used in future developments of anti-*Salmonella* therapy targeting genes involved in these key processes. Such treatment could be a valuable alternative, as the prevalence of antibiotic-resistant bacteria is increasing very rapidly.

## INTRODUCTION

Salmonellosis (either typhoidal or nontyphoidal) is one of the major causes of foodborne diseases globally, affecting over 200 million people and resulting in over 200,000 fatal cases per year (1, 2). Animal salmonellosis is of considerable importance not only because of animal infection but also as a reservoir for human infections (3). The causative agent in both humans and animals is Salmonella enterica subsp. *enterica*, which is comprised of more than 1,500 serovars, of which only a few are of clinical significance (4). Based on clinical presentation and host range, *Salmonella* serovars can be classified as host restricted, host adapted, or host unrestricted (5). Most serovars from clinical samples are host unrestricted and are able to infect multiple hosts, usually resulting in gastroenteritis (6). Host-adapted specialists like *S*. Dublin and S. enterica subsp. *enterica* serovar Choleraesuis mainly cause systemic disease in cattle and pigs, respectively, but can sporadically cause systemic infections in other hosts (e.g., humans) (7, 8). Host-restricted serovars are limited to a single host where they cause systemic infections, for example, *S*. Gallinarum (poultry) or *S*. Typhi (human and primates) (1, 9).

As a facultative intracellular, gastrointestinal pathogen, the initial steps of *Salmonella* pathogenesis involve adhesion to and invasion of host intestinal epithelial cells (10). *Salmonella* has evolved various strategies for adhesion and invasion, and different serovars have developed specific combinations of various adhesins or variants of the same adhesin for host cell attachment (11, 12). Adhesive structures of Salmonella enterica can be divided into three groups: fimbrial adhesins, nonfimbrial adhesins, and atypical adhesive structures (13). The role of the majority of these in the pathogenesis of various *Salmonella* serovars remains unknown. *Salmonella* invasion of host cells is mediated by the type three secretion system (T3SS) located on *Salmonella* pathogenicity island 1 (SPI-1) (14), although additional factors, such as the proteins Rck and PagN, have also been found to play a role (15, 16). Despite an overwhelming amount of research and a seemingly clear picture of the adhesion and invasion process, largely based on work with the serovar *S*. Typhimurium, our understanding of these phenomena is still not complete. For example, it is now evident that adhesion and invasion can be affected by expression of different adhesin variants, mutations in genes encoding T3SS effectors, environmental factors, stress, metabolism, and various regulatory proteins (12, 17). Additionally, around 35 to 40% of bacterial genes have no experimentally validated function, and their contribution to the bacterial virulence, including adhesion and invasion, remains unknown (18). Therefore, the search for new genes involved in these processes is highly desirable.

To find new virulence factors contributing to adhesion or invasion, in this study we investigated the adhesion and invasion of various *Salmonella* serovars against model cell lines derived from three host species: human Caco-2, porcine IPEC-J2, and chicken CHIC-8E11. The study included host-restricted *S*. Gallinarum (from chicken), host-adapted *S*. Dublin (from cattle) and *S*. Choleraesuis (from various animals, largely swine), and host-unrestricted *S*. Typhimurium (from humans and pigs) and *S*. Enteritidis (from humans and chicken). For quantification of adherent and invasive *Salmonella in vitro*, we used a previously established VideoScan technology (19, 20) with a new VideoScan module specifically developed for high-throughput quantification of *Salmonella*-infected cells. Moreover, using whole-genome sequencing, we identified mutations affecting adhesion and invasion in novel genes for which contributions to adhesion and invasion were further validated. Additionally, we used transcriptome sequencing (RNA-seq) to identify the differentially expressed genes affecting adhesion and invasion. This study improves the understanding of mechanisms underlying adhesion and invasion of host epithelial cells during *Salmonella* infection and shows the contribution of small genome alterations to bacterial virulence.

## RESULTS

### *Salmonella* transformation.

Bacterial isolates were transformed with a green fluorescent protein (GFP)-expressing plasmid for automated quantification by our VideoScan method. To do this, susceptibility to ampicillin (Amp) and kanamycin (Kan) was first assessed for 128 *Salmonella* isolates. Most isolates were sensitive to both antibiotics, one isolate was resistant to kanamycin, and 37 were resistant to ampicillin. A single isolate was resistant to both ampicillin and kanamycin and, therefore, was excluded from further work. Due to the high number of ampicillin-resistant isolates, the antibiotic resistance cassette in plasmid pFPV25.1 was exchanged from ampicillin to kanamycin to create plasmid pFPV25.1Kan. The remaining 127 isolates were successfully transformed with this new plasmid.

### Quantification of *Salmonella* with VideoScan.

**FIG 1:**
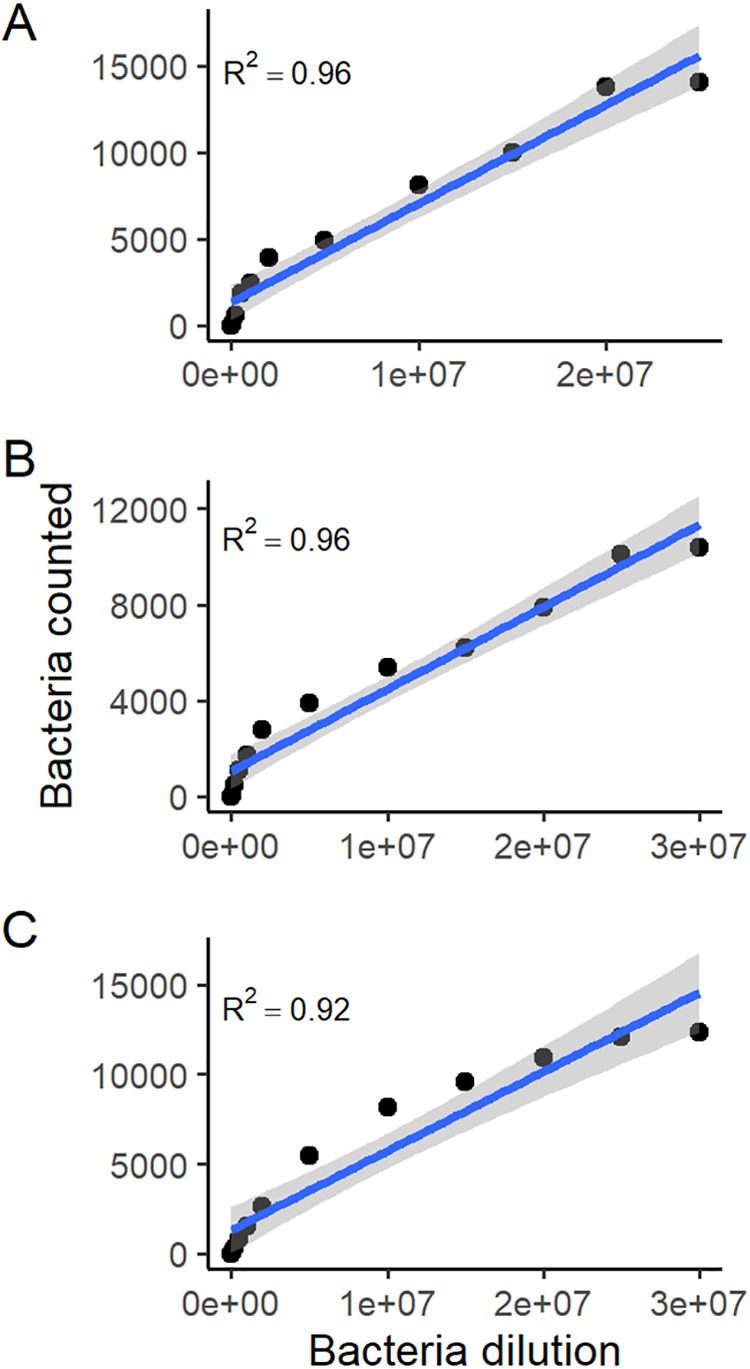
Dilution series of *Salmonella* in cell line infection assays. This assay was performed to determine the linear range of GFP-expressing *S*. Typhimurium SL1344 in infection assays with IPEC-J2 (A), Caco-2 (B), and CHIC-8E11 (C) cell lines in a 96-well plate format for 1 h. Images were automatically taken by the VideoScan instrument. Black dots represent a median value from three assays in triplicates for each bacterial dilution. Blue lines connecting dots are smoothed trend lines, and gray areas around the lines are 0.95 confidence intervals of the linear regression.

Before proceeding to automated quantification of infectivity of the 127 GFP-expressing isolates, the reliability of the fluorescence microscope-based VideoScan method was assessed. Serial dilution experiments were performed with *S*. Typhimurium SL1344 to determine the dynamic range of the VideoScan. The quantification of *Salmonella* was possible within a wide range of bacterial dilutions for all three cell lines tested (Fig. 1). The linear range of the assay was 2 × 10^4^ to 2.5 × 10^7^ (*R*^2^ = 0.96), 2 × 10^4^ to 3 × 10^7^ (*R*^2^ = 0.96), and 2 × 10^4^ to 3 × 10^7^ (*R*^2^ = 0.92) for IPEC-J2, Caco-2, and CHIC-8E11, respectively. Thus, quantification of GFP-labeled *Salmonella* was reliable within the tested serial dilution range. A bacterial concentration of 2 × 10^6^ bacteria/well (4 × 10^7^/ml) was chosen for further assays.

### Cell line infection screening revealed no link between cell type and source of host specialist *Salmonella* isolates.

**FIG 2:**
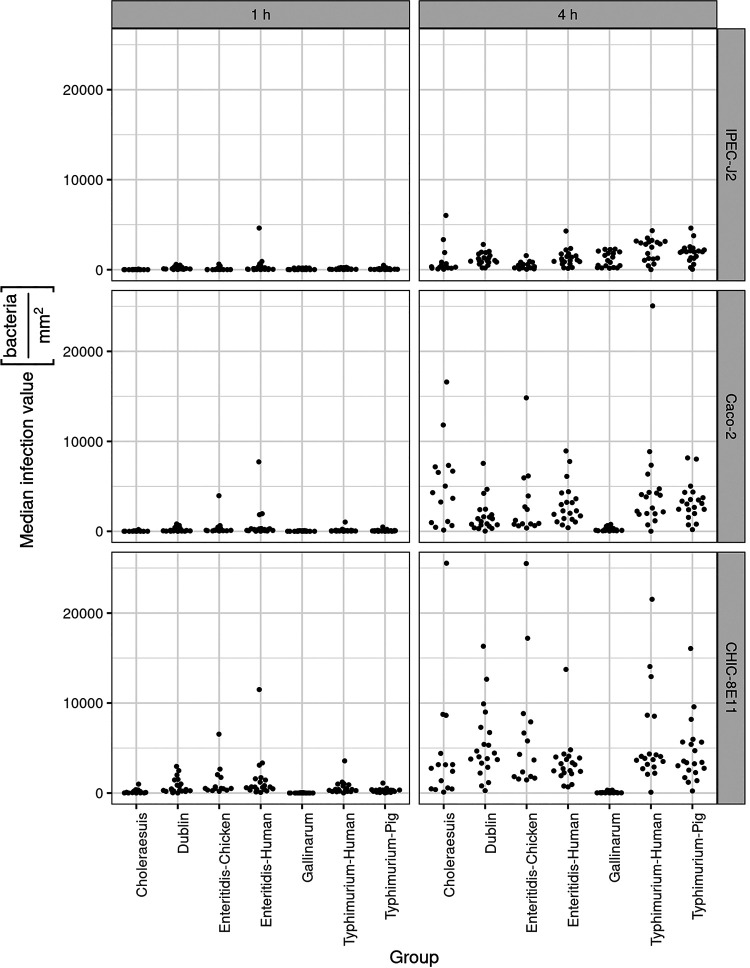
*Salmonella* infection of IPEC-J2, Caco-2, and CHIC-8E11 cell lines. One hundred twenty-seven *Salmonella* isolates from *S*. Typhimurium of human (Typhimurium-human) and pig (Typhimurium-pig) origin, *S*. Enteritidis of human (Enteritidis-human) and chicken (Enteritidis-chicken) origin, and *S*. Choleraesuis (Choleraesuis), *S*. Dublin (Dublin), and *S*. Gallinarum (Gallinarum) were compared for IPEC-J2, Caco-2, and CHIC-8E11 cell line infection after 1 h and 4 h of incubation. Each dot represents a median infection value from three measurements in triplicates for one strain. Results are shown as the number of bacteria per square millimeter.

Porcine (IPEC-J2), human (Caco-2), and chicken (CHIC-8E11) intestinal epithelial cells were infected with the 127 GFP-expressing *Salmonella* isolates for 1 and 4 h. Bacteria that infected cells were counted using the VideoScan, and data were fitted to an appropriate statistical model (Fig. 2).

Surprisingly, *Salmonella* host specialists did not appear to be more infective in cell lines originating from the host species from which they were isolated. *S*. Choleraesuis (pig specialist) showed 12.5 times lower infection rates in porcine IPEC-J2 cells than Caco-2 cells (*P* < 0.01), whereas *S*. Gallinarum (chicken specialists) showed the highest infection in these cells, and this result was 20 times higher than that of infection in CHIC-8E11 cells (*P* < 0.001). *S*. Typhimurium isolated from humans also did not show greater infection of human Caco-2 cells. Similar results were found using *S*. Enteritidis from humans or chicken and *S*. Typhimurium from pigs. Among all *Salmonella* groups, *S*. Gallinarum was 50 to 100 times less infective than other groups on CHIC-8E11 (*P* < 0.001), and *S*. Choleraesuis was 1.2 to 28 times more infective than other groups on Caco-2 (statistically significant only for comparison with *S*. Gallinarum; *P* < 0.001). In general, CHIC-8E11 cells were most susceptible to *Salmonella* infection (*P* < 0.01), while IPEC-J2 cells were most resistant (*P* < 0.01) (see Table S2 in the supplemental material).

When the increase of infecting bacteria was calculated between the two incubation times (ΔInf = Inf_4h_ − Inf_1h_), *S*. Choleraesuis had the highest median increase (1.2- to 33-fold) on Caco-2 cells compared with other groups (statistically significant only for comparison with *S*. Gallinarum, *P* < 0.001) (Fig. S1 and Table S3). The highest ΔInf on CHIC-8E11 cells was observed for *S*. Dublin (1.2- to 78-fold higher, statistically significant for comparison with *S*. Gallinarum and *S*. Enteritidis human, *P* < 0.01) and on IPEC-J2 for *S*. Typhimurium isolated from humans (1.3- to 8.3-fold higher, statistically significant for comparisons with *S*. Choleraesuis, *S*. Dublin, and *S*. Enteritidis from chicken and *S*. Enteritidis from human, *P* < 0.01). Nearly all *Salmonella* groups had the lowest ΔInf in IPEC-J2, with the exception of *S*. Gallinarum, which had the highest ΔInf in this cell line (*P* < 0.01).

### Analysis of *Salmonella* genomes revealed the presence of natural mutations responsible for altered infection phenotypes.

**FIG 3:**
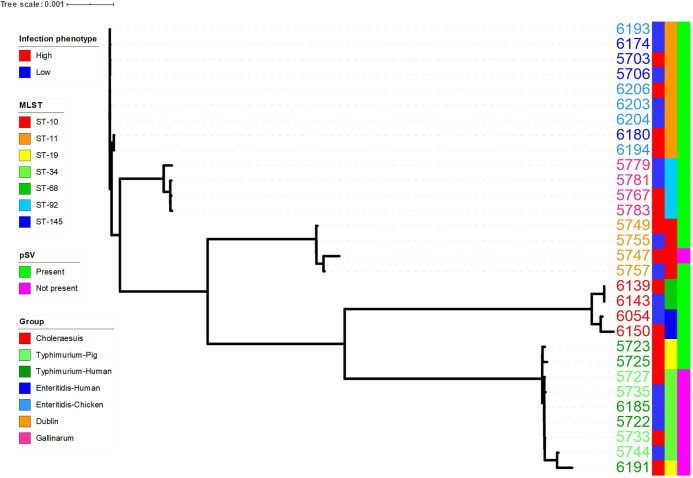
Core genome tree of *Salmonella* with different infection phenotypes. Annotated genomes were analyzed with Roary. Next, the core genome alignment of 3,473 genes from 30 genomes was used to generate a tree with FastTree 2.1. Information about infection phenotype, group, MLST sequence types, and presence of pSV plasmid was added with the use of iTOL. Groups and isolate numbers used in the analysis are the following: Choleraesuis, 6054, 6139, 6143, and 6150; Typhimurium-pig, 5727, 5733, 5735, and 5744; Typhimurium-human, 5722, 5723, 5725, 6185, and 6191; Enteritidis-human, 5703, 5706, 6174, and 6180; Enteritidis-chicken, 6193, 6194, 6203, 6204, and 6206; Dublin, 5747, 5749, 5755, and 5757; Gallinarum, 5767, 5779, 5781, and 5783.

To identify bacterial factors responsible for various levels of infectivity, the genomes of 30 isolates with the highest and lowest infectivities were sequenced and compared. The pangenome analysis revealed no differences in gene content (i.e., gene presence or absence) associated with infection phenotype between highly infective isolates compared to marginally infective isolates. To determine the similarity among isolates with altered infection phenotypes, core genome-based and additional binary genome-based phylogenetic trees were constructed (Fig. 3 and Fig. S2). Moreover, multilocus sequence typing (MLST) sequence types (ST) and the presence of pSV plasmids that are common in almost all serovars were determined. Highly infective isolates did not form separate subclades/groups and did not belong to different STs. The presence of pSV plasmids did not correlate with infectivity levels.

All the results at this stage of work lead to the hypothesis that altered infection phenotypes were driven not by gene content but by sequence variation in *Salmonella* genomes. Therefore, sequencing reads were mapped to reference genomes and aligned reads were compared to identify any gene mutations compared to the reference genome. Fourteen candidate genes were found to be potentially associated with altered infection phenotypes and are listed in Table 1 with function, description of mutation, and whether similar mutations could be found in genomes in the GenBank database. The functions of these 14 candidate genes were (i) virulence gene expression regulation (e.g., *dksA*), (ii) synthesis of cell wall or membrane elements (e.g., *rfaL*), and (iii) cell membrane components (e.g., *ompD*). The role of several of these genes in *Salmonella* adhesion and invasion of cells has not been investigated so far (e.g., *sanA* and *yidR*). Natural mutations found in the strains with altered infection phenotypes included deletions and single-nucleotide substitution, which resulted in premature stop codons or amino acid alterations. Alleles with these natural mutations were found for six genes in the GenBank reference genome database with BLASTN search.

**TABLE 1:** Candidate genes responsible for altered infection phenotype with a description of their function (where possible), mutation, and presence of genomes with the same changes in the GenBank database^*a*^

Serovar	Gene	Function^*b*^	Mutation^*c*^	GenBank query (day/mo/yr)
*S*. Enteritidis	*dksA*	Required for growth in minimal medium and intestinal colonization; mediates adaptation to oxidative and nitrosative stress	Two aa deletions at positions 87 and 88 in isolate 5706	Yes (54 sequences, 14 sequences with mutations in the same region)
*S*. Enteritidis	*ompD-nmpC*	Porin OmpD, mediates binding of Tym to T84 and U937 cells	Deletion of starting 300 bp of CDS in isolate 6174	No
*S*. Enteritidis	*yidR*	Putative ATP/GTP-binding protein; mutant has reduced binding to/persistence in lettuce	Stop codon at position 317 in isolate 6174	Yes (4 genomes, 21/10/2019)
*S*. Enteritidis	*sanA*	Vancomycin high-temp exclusion protein; required for bile tolerance in *S*. Typhi	Deletion of 10 nt in isolate 6203	Yes (1 genome, 14-nt deletion in the same region, 21/10/2019)
*S*. Enteritidis	*rfaL* (*waaL*)	O-antigen ligase; in Tym, affects motility	12-nt deletion in isolate 6193	No (21/10/2019)
*S*. Typhimurium	*sirA*	Invasion response regulator; global regulator of genes mediating enteropathogenesis	14-nt deletion in isolate 5735	Yes (2 genomes, 21/10/2019)
*S*. Typhimurium	*aroA*	3-Phosphoshikimate 1-carboxyvinyltransferase; mutation leads to auxotrophy for aromatic amino acids	78-nt deletion in isolate 6185	No (21/10/2019)
*S*. Typhimurium	*fimH*	Adhesin of type 1 fimbriae	Stop codon at position 80 in isolate 6191	Yes (13 genomes, 21/10/2019)
*S*. Gallinarum	*lrhA*	Transcriptional regulator; negative regulator of flagellum expression in early cell growth phases in Tym	3-nt deletion (at 100 position), 1 aa (Ala), in isolate 5781	Yes (7 genomes, 21/10/2019)
*S*. Gallinarum	*rcsD*	Phosphotransfer intermediate protein in a two-component regulatory system with RcsBC; implicated in the control of capsule and flagellum synthesis and biofilm formation	5-nt deletion in isolate 5781	No
*S*. Dublin	*ompW*	Outer membrane protein W; mediates methyl viologen (paraquat) efflux in Tym	1-nt deletion in isolates 5755 and 5757	No
*S*. Dublin	*mpl*	UDP-N-acetylmuramate:l-alanyl-gamma-d-glutamyl-meso-diaminopimelate ligase; peptidoglycan synthesis	Deletion after codon 423 in isolate 5757	No
*S*. Choleraesuis	*rtsA*	Increases expression of the invasion genes by inducing *hilA*, *hilD*, and *hilC* genes and the *invF* operon	1 SNP that leads to change from Arg to Gln in isolate 6143	No
*S*. Choleraesuis	*wza*	Polysaccharide (colanic acid) export protein Wza; involved in exopolysaccharide production	1 SNP in codon 171 that leads to change for stop codon in isolate 6054	No

a GenBank query was made with the use of the RefSeq Genomes Database.

b Tym, *S*. Typhimurium.

c aa, amino acid; CDS, coding sequence.

### Functional assays confirm the role of *dksA* and *sanA* in host cell infection.

To confirm the contribution of mutations to altered infection phenotypes, two genes were selected and genetically manipulated for further elucidation of their functions: *dksA* and *sanA* (the gene functions are summarized in Table 1). To study the effect of mutations found in this work, *dksA* and *sanA* in *S*. Enteritidis clinical isolates (no. 5706 and 6203, respectively) were replaced with the wild-type sequences from *S*. Enteritidis PT4 strain no. P125109. No obvious phenotypic changes compared to parental strains were observed in the complemented strains, indicating that the genetic manipulations did not affect bacterial morphology. Isogenic strains of isolates 5706 and 6203 were tested for *dksA* and *sanA* expression, respectively. Relative expression of wild-type *dksA* was 40% lower than that of the *dksA* mutated variant (*P* = 0.001). For 6203 isogenic strains, there was no difference in expression between wild-type and mutated *sanA* variants.

**FIG 4:**
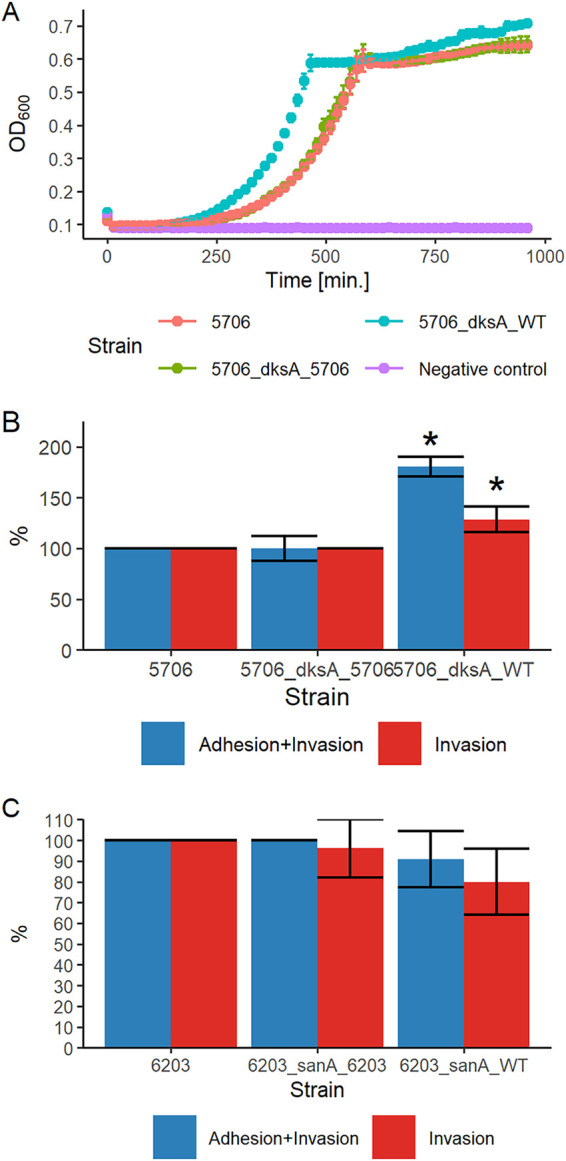
Phenotypic assays for *dksA* and *sanA* genes. (A) Growth curve of *S*. Enteritidis 5706 isogenic strains in M9 minimal medium supplemented with 0.2% glucose. Dots represent median values from three assays in triplicates for each time point. (B) Gentamicin protection assay with the use of *S*. Enteritidis 5706 isogenic strains and Caco-2 cells. Strains used in panels A and B are the following: 5706, isolate with the mutated *dksA* variant; 5706_dksA_5706, isolate with the mutated *dksA* variant introduced by negative selection system; 5706_dksA_WT, isolate with wild-type *dksA* introduced by negative selection system. (C) Gentamicin protection assay with the use of *S*. Enteritidis 6203 isogenic strains and Caco-2 cells. 6203, isolate with the mutated *sanA* variant; 6203_sanA_6203, isolate with the mutated *sanA* variant introduced by negative selection; 6203_sanA_WT, isolate with wild-type *sanA* introduced by negative selection. Error bars represent the median absolute deviation. Statistically significant results in panel B are marked with an asterisk (*P* = 0.05).

Gene *dksA* encodes a stringent response regulator required for growth in minimal medium; therefore, the isogenic strains of isolate 5706 were tested for growth in M9 minimal medium supplemented with glucose. The strain expressing wild-type DksA had 1.7- to 1.9-fold higher growth rate and doubling time in exponential phase of growth than the strains expressing a DksA mutant variant (*P* < 10^−5^) (Fig. 4A and Table S4). Subsequently, the ability to adhere to and invade Caco-2 cells was evaluated, and the wild-type strain had 80% higher infection and 30% higher invasion levels than the strains with a *dksA* mutant variant (*P* = 0.05) (Fig. 4B).

The *sanA* gene deletion in Escherichia coli has been linked to increased susceptibility to vancomycin. The function of this gene is often studied *in vitro* by vancomycin susceptibility testing. Therefore, isogenic strains of isolate 6203 with the wild-type and mutated forms of *sanA* were tested for growth in vancomycin. Strains carrying the mutated variant were more resistant to vancomycin than strains carrying wild-type *sanA*. Strains with the wild-type *sanA* gene showed growth in up to 31.25 μg/ml vancomycin, whereas strains with a mutated *sanA* survived concentrations up to 62.5 μg/ml. Next, strains carrying isogenic variants of *sanA* were tested in adhesion and invasion assays. Strains carrying a mutated variant of *sanA* infected cells at a 10% higher level and invaded at a 20% higher level than the strain carrying the wild-type *sanA* gene, but the statistical analysis revealed that these differences are not significant (Fig. 4C).

### Different expression levels of T3SS are responsible for altered infection phenotypes in *S*. Typhimurium and *S*. Enteritidis isolates.

**FIG 5:**
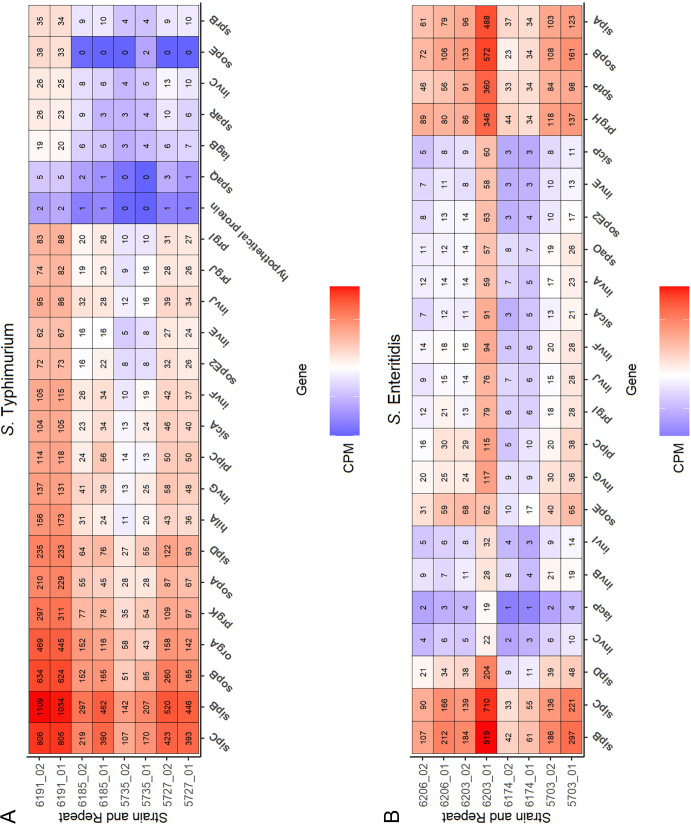
Differentially expressed genes associated with epithelial cell invasion. Heatmap with expression levels of genes associated with invasion of epithelial cells in four *S*. Typhimurium (A) and four *S*. Enteritidis (B) isolates. Strain number and repeat are shown on the *y* axis. Isolates 5703, 5727, 6191, and 6206 represent high-infectivity cell line phenotypes, while isolates 5735, 6174, 6185, and 6203 represent low-infectivity phenotypes. Names of DE genes are shown on the *x* axis. Color gradient is proportional to counts per million (CPM) for each gene in each repeat. The numbers of CPM for each gene and repeat are written in rectangles.

Based on the results from the RNA-seq and transcriptome studies, the number of differentially expressed (DE) genes between isolates varied from 15 to 67 in the case of *S*. Enteritidis and from 48 to 324 for *S*. Typhimurium isolates (Table S5). All DE genes were grouped by biological processes they represent, and this analysis revealed the presence of two gene groups, “bacterial secretion system” and “bacterial invasion of epithelial cells,” associated with invasion of epithelial cells (Fig. S3A and B). These genes belong to the T3SS injectosome machinery or are secreted into epithelial cells and mediate bacterial internalization. Two *S*. Typhimurium isolates representing the high-infectivity phenotype (isolate no. 5727 and 6191) had from 2- to 280-fold increased expression of these genes compared to two isolates with a low-infectivity phenotype (isolate no. 5735 and 6185) (Fig. 5). This was not the case for *S*. Enteritidis, as one *S*. Enteritidis isolate (isolate no. 6203) with low-infectivity phenotype had expression profiles similar to those of two *S*. Enteritidis isolates with high-infectivity phenotypes, indicating a contribution of other factors to the adhesion and invasion process.

## DISCUSSION

This study was aimed at identifying new genes with possible contributions to adhesion and/or invasion by *Salmonella* with host epithelial cells. To develop a high-throughput screening method for the quantification of *Salmonella* in cell culture infections, we used the VideoScan platform developed in our laboratory for nucleic acid detection or quantification, E. coli quantification in cell culture adhesion assays, quantification of bacterial adhesion to proteins, and automated detection of autoantibodies in cell-based assays (21–24). The new VideoScan module leverages the detection of and screening of adherent and invasive GFP-expressing bacteria to provide an output of bacterial numbers adhering to and invading a cell line. This assay has advantages over other bacterial quantification methods in that it is simpler, avoids complicated staining procedures involving membrane permeabilization, avoids the often laborious selection of suitable antibodies for different serovars and problems arising from nonspecific antibody binding, and does not require the performing of dilution series and CFU number determinations on agar plates (25). The only disadvantage of this approach is the requirement for bacterial transformation with a plasmid encoding GFP, where it could be difficult to find an antibiotic selection marker for antibiotic-resistant isolates (26).

This approach was used to test if 127 *Salmonella* isolates, representing serovars with different host ranges, were better at infecting cells originating from the associated host. However, using this model, this was shown not to be the case. This might be because there are many other host factors related to *Salmonella* host specificity that were not present in our simple model of epithelial cell infection (27).

In an effort to explain the differences in infectivity among isolates, we focused our efforts on analyzing and identifying the possible underlying genetic explanations for the outcomes in the cell line infection assays in isolates that showed altered infection phenotypes compared to the majority of isolates screened in each group. Although the *Salmonella* virulence plasmid (pSV) was previously associated with septicemia and enteritis (28), we were not able to find any association between altered infection phenotype and the presence of pSV. However, we were able to find naturally occurring mutations present in the genomes of clinical isolates of *Salmonella*. Interestingly, the majority of the mutations we found were not associated with any factors directly responsible for invasion or adhesion. We did not identify any mutations in genes encoding T3SS, Rck, or PagN that would explain the altered infection phenotypes or in genes encoding adhesins, except for a single base substitution in the *fimH* gene leading to premature translation termination in one of the isolates with a high-infectivity phenotype. Usually, loss of expression of type 1 fimbriae (T1F) (the *fimH* gene codes for the tip adhesin of T1F), which this deletion confers, results in lower cell adhesion (29). It is noteworthy that bacteria for adhesion assays with T1F are usually grown under conditions different from those used in our screens (12, 30).

When we looked at the function of genes with natural mutations, we noticed that they are related to cell membrane/surface structures and cell wall or surface structure expression regulation. There are reports that link pseudogene formation in cell membrane/surface structures with host adaptation (31, 32). These could explain the presence of natural mutations in host-restricted (*S*. Gallinarum) and host-adapted serovars (*S*. Dublin and *S*. Choleraesuis). Other explanations for natural mutations could be that these isolates were isolated from asymptomatic carriers, and the presence of natural mutations might increase long-term survival within the host without recognition by the immune system. Similar findings were ascertained by Klemm et al., where the formation of pseudogenes in membrane proteins has been reported in *S*. Enteritidis isolates of one strain sampled multiple times over 15 years from one patient (33). Unfortunately, we had no information about the host’s clinical status at the time of sampling; therefore, we cannot confirm or rule out this possibility.

Another possibility that could explain the occurrence of altered infection phenotypes and natural mutations is the mutagenic properties of antibiotics or other xenobiotics. Multiple studies have shown that various classes of antibiotics can induce stress and mutations at subinhibitory concentrations (34–36). These mutations are the effect of adaptive bacterial response to antimicrobials or a consequence of direct interaction of antibiotics with bacterial DNA (37). When we checked for the presence of antibiotic resistance genes, nine out of 30 *Salmonella* isolates had at least one antibiotic resistance gene, confirming a possible exposure to antibiotics. Antibiotic-induced mutations have a deleterious effect, such that the bacteria bearing them would have decreased fitness and more negative selection compared to wild-type isolates. This negative selection perhaps could explain why the natural mutations that we observe in our strain collection can rarely be found in GenBank. To confirm this hypothesis in the future, an antibiotic-driven mutant library could be created and tested for infection of epithelial cells.

Contribution of mutations to altered infection phenotype was functionally validated for two genes: *dksA* and *sanA*. Gene *dksA* was selected because its contribution to host cell adhesion and invasion was known. Little was known about the influence of *sanA* on *Salmonella* host cell adhesion; therefore, it was chosen for further investigation. Complementation of the mutation found in *dksA* led to an increase in growth rate in minimal medium and host cell infectivity. Although the importance of the amino acid at position 88 in DksA for growth in minimal medium has been described previously (38), a link to cell line invasion has not been described so far. Deletion of *sanA* was previously associated with increased susceptibility to vancomycin in E. coli (39). One of our isolates with altered infection phenotypes had a 10-nucleotide (nt) deletion in *sanA*. Therefore, we exchanged the mutation with the wild-type sequence and determined whether this would lead to the rescue of certain functions. Indeed, complementation of the mutation led to a decrease in resistance to vancomycin and further influenced adhesion and invasion. Although the mode of action for *dksA* in adhesion and invasion was described for *S*. Typhimurium in previous studies (38, 40, 41), there is no information about the role or possible contribution of *dksA* sequence variation and *sanA* to bacterial adhesion or invasion.

We decided to get a global overview of expression profiles for the isolates with altered infection phenotypes, so we conducted a transcriptomic analysis, which highlighted the importance of T3SS expression levels in the determination of an isolate’s infection phenotype. Similar differences were observed in the study of Shah, where 38 genes from SPI-1 showed reduced expression in less-virulent *S*. Enteritidis isolates than in more-virulent *S*. Enteritidis (42). Three isolates with low-infectivity phenotypes included in this analysis had mutations in *aroA*, *ompD*, *yidR*, and *sirA*. Although the contribution of *aroA* and *sirA* to the regulation of genes associated with the T3SS has been shown previously and there is information about a possible connection between T3SS and *ompD* expression levels (43–45), so far there is no information about the *yidR* gene. One strain with a low-infectivity phenotype and a mutation in the *sanA* gene had a gene expression profile similar to that of the high-infectivity phenotype. Since knowledge about this gene is scant, an explanation for this observation requires further study.

Our analysis of genomes and transcriptomes of the isolates with altered infection phenotypes allowed us to find explanations for lowered infection of epithelial cells. However, we were not able to identify the factors contributing to high-infectivity phenotypes of the selected *Salmonella* isolates. It is possible that other factors, like methylation, influence higher virulence of these isolates, as shown before in the case of *Salmonella* (46). A detailed investigation of these additional factors is out of the scope of the present investigation.

To summarize, this work was an exploration of what we can learn from observing the interaction of clinical isolates of *Salmonella* with host cells of intestinal epithelial origin. We have shown that the majority of isolates have similar infection phenotypes, and isolates with altered infection phenotypes provide a source for determination of new genes or new gene variants influencing epithelial infection, including novel factors associated with adhesion and invasion of epithelial cells by *Salmonella*. We identified two genes, *sanA* and *yidR*, that were not previously associated with adhesion or invasion of host cells. We consider these genes to be interesting candidates for further investigations within the context of *Salmonella* virulence. Our study reveals new questions regarding the origin of such mutations and their contribution to pathogen evolution and pathogenicity, and it suggests that the approach used here, with larger strain collections belonging to the same serovar, clinical data, and the use of genome-wide association studies, provides important insights into *Salmonella* host-cell interactions.

## MATERIALS AND METHODS

### Bacterial isolates and plasmids.

All bacterial isolates used in this study (Table 2) were tested for ampicillin (Amp) and kanamycin (Kan) resistance with agar disc diffusion tests. For ampicillin-sensitive isolates, plasmid pFPV25.1, containing an Amp resistance gene and the GFPmut3 fluorescent protein expressed from the constitutive *rpsM* promoter, was used (47). To exchange the Amp resistance gene with a Kan resistance gene for those isolates that were ampicillin resistant but kanamycin sensitive, Gibson assembly reaction was performed (NEB). The primers that were used for the amplification of pFPV25.1 plasmid and Kan resistance cassette are listed in Table 3. PCR was performed with the use of Phusion high-fidelity DNA polymerase (Thermo Scientific) according to the manufacturer’s protocol. Gibson assembly was performed according to the manufacturer’s protocol. The new plasmid was named pFPV25.1Kan.

**TABLE 2:** Bacteria used in this study

Strain	Description (group name)	Isolate reference no.	Source
Escherichia coli XL1Blue	*recA1 endA1 gyrA96 thi-1 hsdR17 supE44 relA1 lac* [F*´ proAB lacI*^q^*ZΔ*M15 Tn*10* (*tetR*)]		Agilent Technologies
Salmonella enterica subsp. *enterica* serovar Typhimurium	20 strains, isolated from human diarrhea cases (Typhimurium-human)	5717–5725, 6181–6191	Mydlak/Thorausch Diagnostic Laboratory, Cottbus, Germany
Salmonella enterica subsp. *enterica* serovar Typhimurium	20 strains, isolated from porcine feces samples (Typhimurium-pig)	5726–5745	German Federal Institute for Risk Assessment, Berlin, Germany
Salmonella enterica subsp. *enterica* serovar Enteritidis	20 strains, isolated from human diarrhea cases (Enteritidis-human)	5702–5710, 6170–6180	Mydlak/Thorasch Diagnostic Laboratory, Cottbus, Germany
Salmonella enterica subsp. *enterica* serovar Enteritidis	14 strains, isolated from chicken feces samples (Enteritidis-chicken)	6192–6195,6197, 6198, 6200–6207	German Federal Institute for Risk Assessment, Berlin, Germany
Salmonella enterica subsp. *enterica* serovar Gallinarum	19 strains, isolated from chicken feces samples (Gallinarum)	5766–5771, 5773–5785	German Federal Institute for Risk Assessment, Berlin, Germany
Salmonella enterica subsp. *enterica* serovar Dublin	20 strains, isolated from bovine feces samples (Dublin)	5746–5765	German Federal Institute for Risk Assessment, Berlin, Germany
Salmonella enterica subsp. *enterica* serovar Choleraesuis	15 strains, isolated from various sources, i.e., porcine feces, wild boars, meat products, reptile (Choleraesuis)	6053, 6054, 6138–6150	German Federal Institute for Risk Assessment, Berlin, Germany
Salmonella enterica subsp. *enterica* serovar Typhimurium SL1344			Free University, Berlin, Germany

**TABLE 3:** Primers used in this study

Primer name	Primer sequence (5′–3′)	Gene	Reference or source
pFVP25.1KmRev	CTGTCAGACCAAGTTTACTCATATATAC	*kanR*	This study
KanpFVP25Fwd	TGAGTAAACTTGGTCTGACAGTCAGAAGAACTCGTCAAGAAG	*kanR*	This study
KanpFVP25Rev	TAATATTGAAAAAGGAAGAGTATGATTGAACAAGATGGATTG	*kanR*	This study
pFVP25.1KmFwd	ACTCTTCCTTTTTCAATATTATTG	*kanR*	This study
dksAdelNEGfor	ATGCAAGAAGGGCAAAACCGTAAAACATCGTCCCTGAGTATTCTCGCCATGCTTGCAGTGGGCTTACATG	*dksA*	This study
dksAdelNEGrev	TTAACCCGCCATCTGTTTTTCGCGAATTTCAGCCAGCGTTTTGCAGTCGATCAGAGCAGGATCGACGTCC	*dksA*	This study
dksAfor	ATGCAAGAAGGGCAAAACCG	*dksA*	This study
dksArev	TTAACCCGCCATCTGTTTTTCG	*dksA*	This study
dksA100UpstreamFor	ACAGGGTTGTCAAGTGTTACG	*dksA*	This study
dksA100DownstreamRev	TAACGAGCCGAAATGCAGTTC	*dksA*	This study
dksAinternalRev	AGTGCGATCGACTTCATCC	*dksA*	This study
k2	CGGTGCCCTGAATGAACTGC	*kanR*	68
sanAdelNEGfor	ATGTTAAAGCGCGTGTTTTACAGCCTGTTGGTCCTGGTAGGCTTGCTGCTGCTTGCAGTGGGCTTACATG	*sanA*	This study
sanAdelNEGrev	TCATTTCCCTTTTTTCTTTTCCAGTTCAAGCAATTGTTCCGGCGTAACTGTCAGAGCAGGATCGACGTCC	*sanA*	This study
sanAfor	ATGTTAAAGCGCGTGTTTTAC	*sanA*	This study
sanArev	TCATTTCCCTTTTTTCTTTTCCAG	*sanA*	This study
sanA100UpstreamFor	CGATACAAGGGAAATCATGCTG	*sanA*	This study
sanA100DownstreamRev	TTCCAGGCCTCACGGAAG	*sanA*	This study
sanAinternalRev	GCCCTGGATACGATAACGA	*sanA*	This study
tufAqPCRfor	TGTTCCGCAAACTGCTGGACG	*tufA*	69
tufAqPCRrev	ATGGTGCCCGGCTTAGCCAGTA	*tufA*	69
dksAqPCRfor1	TGAAGCATGGCGTAATCAACTC	*dksA*	This study
dksAqPCRrev1	TCCAGGCTAAACTCCTCTTCC	*dksA*	This study
sanAqPCRfor1	AAACAGCGCCCTATATCTATGAC	*sanA*	This study
sanAqPCRrev1	TGATTAATGACACCCTTGCGA	*sanA*	This study

For adhesion and invasion screening, all 127 *Salmonella* isolates were GFP labeled by transformation with pFPV25.1 or pFPV25.1Kan. Electrocompetent bacteria were prepared as described previously (70), with some modifications. After transformation with plasmid, single colonies were restreaked on fresh Luria-Bertani (LB) agar plates (with appropriate antibiotics). Cultures were prepared in 1 ml of LB (with appropriate antibiotics) in 2-ml Eppendorf tubes and grown for 16 h, and glycerol-based cryostocks were prepared from these cultures.

### Cell culture.

Three intestinal epithelial cell lines were used in this study: porcine IPEC-J2 (obtained from Peter Schierack [48]), human Caco-2 (DMSZ, Germany), and chicken CHIC-8E11 (MicroMol GmbH; obtained from Karsten Tedin, FU Berlin). Cells were grown in Dulbecco’s modified Eagle’s medium (DMEM)/Ham’s F-12 (Millipore) supplemented with 1 mM l-glutamine and penicillin-streptomycin (Millipore) and either 5% (IPEC-J2 and CHIC-8E11) or 10% (Caco-2) bovine serum (Millipore) and were incubated at 37°C with 5% CO_2_ and passaged using standard protocols. For infection assays, IPEC-J2, Caco-2, and CHIC-8E11 cells were seeded in Nunclon Delta 96-well plates (Nunc) at a density of 0.7 × 10^4^, 1 × 10^4^, and 2 × 10^4^, respectively, and used after 5 days. For the gentamicin protection assay, Caco-2 cells were seeded in 24-well plates (Nest) at a density of 5.8 × 10^4^ cells and used after 5 days.

### VideoScan module for cell line infection assays.

For automated bacterial quantification, we further developed our VideoScan technology (21) by creating a new module. Briefly, the VideoScan hardware consists of an inverse epifluorescence Olympus microscope with multiple fluorescence channels, a digital camera, and a motorized scanning stage. The VideoScan module works with a ×20 magnification objective and first focuses on 4′,6-diamidino-2-phenylindole (DAPI)-stained cell nuclei in a well, which is then marked as position 0 nm. Afterwards, the software takes a Z-stack of images (for IPEC-J2 and CHIC-8E11, 6 images; Caco-2, 7 images) of bacteria (GFP stained) with a starting position of −2000 nm (for IPEC-J2 and CHIC-8E11) or 0 nm (for Caco-2). Next, one composite image is made from the Z-stack images taken, and bacteria on the image are detected and counted. Twenty composite images per well were analyzed during readout.

### Determination of the linear range of the VideoScan assay.

To test the reliability of our automated bacterial quantification assay, *S*. Typhimurium SL1344, transformed with a pFPV25.1Kan plasmid, was grown to late exponential phase (optical density at 600 nm [OD_600_] of 0.95 to 1.0; 2.85 × 10^8^ to 3 × 10^8^ of bacteria/ml) at 37°C and 180 rpm and washed with phosphate-buffered saline (PBS). After this, 50 μl of bacteria was applied in a dilution series (2 × 10^4^ to 3 × 10^7^/well; final concentration of bacteria, 4 × 10^5^ to 6 × 10^8^/ml) on plates with a monolayer of IPEC-J2, Caco-2, and CHIC-8E11 cells. Bacteria were incubated for 1 h, the plates were washed, and the underlying cell line monolayer with bound bacteria was fixed with 4% paraformaldehyde (PFA; Roth). Cell nuclei were stained with DAPI. Next, adherent bacteria in the plates were counted using the VideoScan module. Three independent experiments with three repetitions for each dilution were prepared and measured.

### Cell line infection assays.

To screen the 127 *Salmonella* isolates for infectivity, cell lines were seeded in 96-well plates and assayed on days 5, 6, and 7 after seeding. A total of 127 *Salmonella* isolates with GFP expression were used for infection assays. *S*. Typhimurium SL1344 was used as a standard strain in all infection assays. Bacteria were grown in 1 ml of LB (with 50 μg/ml Kan or 100 μg/ml Amp) in 2-ml Eppendorf tubes at 37°C and 180 rpm. Before an assay, bacteria were diluted to a concentration of 4 × 10^7^ bacteria/ml, and 50 μl of bacteria was applied per well to a monolayer of IPEC-J2, Caco-2, and CHIC-8E11 cells. Bacteria were incubated for 1 h and 4 h in a cell culture incubator, and the plates were washed three times with PBS. Cell lines with bound bacteria were fixed with 4% PFA in PBS, and the plates were washed with PBS and cell line nuclei were stained with DAPI. Afterwards, adherent bacteria were counted with the VideoScan. Three independent experiments with three repetitions for each *Salmonella* isolate were performed.

### Next-generation genome sequencing and comparative genome analysis.

Genomic DNA was isolated with the Wizard genomic DNA purification kit (Promega) according to the manufacturer’s instructions. DNA was sequenced with the use of the HiSeq X platform at the Sanger Institute Sequencing Facility. Sequencing quality was assessed on the basis of average base quality, GC content, and adapter contamination (49). Genomes were assembled using the Shovill pipeline and assembly_improvement pipeline (50). Genome assemblies were annotated with Prokka (51). Pangenomes were determined by using Roary (52). Core genome phylogeny was performed with FastTree 2.1 software using a generalized time-reversible model of nucleotide evolution. FastTree 2.1 produces results similar to those of maximum likelihood analysis, and the accuracy and reproducibility of this method has been estimated (53). Phylogenetic trees were annotated with iTOL software (54). Comparison of gene presence between isolates with different infection phenotypes was performed with Scoary (55). Multilocus sequence types were determined with the MLST 2.1 online tool, and the presence of pSV plasmid was checked with PlasmidFinder (56, 57).

For single-nucleotide polymorphism search, the reads obtained for each isolate were mapped against a reference genome using bwa (58) (see Table S1 in the supplemental material). Mapped reads were individually analyzed with Artemis (59). All isolates belonging to the same serovar were compared with a reference genome from the same serovar. The mutation found in each genome was considered potentially associated with altered infection phenotypes if it was a deletion or single-nucleotide substitution that resulted in premature stop codons and/or was placed in the gene with confirmed contribution to adhesion or invasion.

### Bacterial mutant construction.

For *S*. Enteritidis clinical isolates 5706 and 6203, a scarless and markerless negative selection-based system was utilized to cure the identified mutations of interest, as in the publication of Khetrapal et al. (60). Primers used for mutant generation are listed in Table 3. To determine whether the isolates showed altered morphologies compared to the parental isolates, cultures (grown for 16 h) were stained with acridine orange and investigated with the use of a fluorescence microscope.

### Growth curves.

Overnight (O/N) cultures (grown for 16 h) or exponential growth cultures were diluted to 5 × 10^6^ bacteria/ml in LB, antibiotic-free cell culture medium, or M9 minimal medium with 0.2% glucose (for strain 5706), and 200 μl of bacterial suspension was pipetted into each well of a 96-well polystyrene plate. Bacterial growth was measured with a Tecan plate reader for 16 h with 30 s of vigorous shaking and OD_600_ measurement every 15 min. Three technical and biological repetitions were performed for each strain. Negative controls were included for each medium used in each assay. To assess differences in growth of 5706 isogenic strains, the package Growthcurver was used, and growth rate (*r*), doubling time (t_gen), and empirical area under the curve (AUC) were used for comparisons between strains (61).

Growth with vancomycin for strain 6203 was performed with the use of O/N cultures (grown for 16 h). Bacteria were diluted in Müller-Hinton broth (MHB; Roth) to a concentration of 1 × 10^6^ bacteria/ml. Twofold serial dilutions of vancomycin in 50 μl of MHB were prepared in 96-well polypropylene plates with the starting concentration of vancomycin set to 1 mg/ml. Afterwards, 50 μl of bacterial suspension was pipetted into each well. Bacterial growth was measured with the use of a Tecan plate reader for 16 h with 30 s of vigorous shaking and OD_600_ measurement every 15 min. For each strain, 3 technical and biological repetitions were performed. No-antibiotic and negative controls were included in each assay.

### RNA isolation and real-time qPCR.

Overnight cultures of strains 5706 and 6203 and their isogenic mutants were grown to an OD_600_ of 0.45 to 0.55. Next, 1 ml of bacteria was harvested and RNA was isolated by the RNAsnap method (62). For each sample, 1 μg of RNA was digested with DNase I (Thermo Scientific) according to the manufacturer’s instructions, and reverse transcription was performed with the use of an iScript cDNA synthesis kit (Bio-Rad). Quantitative PCR (qPCR) was performed with the use of universal SYBR green qPCR supermix (Bio-Rad) and a CFX96 thermocycler (Bio-Rad). Primers used for qPCR are listed in Table 3. The qPCR data analysis was performed with CFX Manager 3.1 (Bio-Rad) by normalizing the expression of *dksA* and *sanA* genes to the reference gene *tufA* with the ΔΔCq method (63).

### Gentamicin protection assays.

Adhesion and invasion assays were performed on strains generated in “Bacterial mutant construction,” above. We chose isolates with an altered infection phenotype. Here, we compared isolates with high infectivity (one phenotype) with isolates with low infectivity (another phenotype) within one serovar. As such, altered infection phenotypes were similarly identified for Caco-2, IPEC-J2, and CHIC-8E11 cells, and further work was done only with the Caco-2 cell line.

All bacteria were previously determined to be susceptible to gentamicin at a concentration of 50 μg/ml. First, O/N cultures of bacteria (grown for 16 h) in 1 ml of LB in 2-ml Eppendorf tubes were diluted to an OD_600_ of 0.05 in 5 ml of LB in 15-ml Falcon tubes and grown to an OD_600_ of 2.0. Next, bacteria were washed once in PBS and resuspended in antibiotic-free cell culture medium. Bacteria (1.2 × 10^7^) in 1 ml of medium was added to Caco-2 cells in each well (proportional to the concentration of bacteria in infection assays in a 96-well plate format). After 1 h of incubation of bacteria with cells, the bacterial suspension was discarded, cells were washed three times with 1 ml of PBS, and new medium with or without gentamicin was added. After 1 h of incubation, supernatants were discarded and cells were washed three times with PBS. Cell monolayers were lysed with 1% Triton X-100 (Sigma) in PBS, dilution series in PBS were made, and bacteria were spread on LB agar plates. The next morning, the number of CFU was counted. For each strain, at least 3 technical and biological repetitions were performed.

### RNA sequencing and differential transcriptome analysis.

Four *S*. Typhimurium and four *S*. Enteritidis isolates with lowest and highest infectivities were selected for RNA sequencing. Overnight cultures were diluted 100× in 10 ml of LB in 50-ml Falcon tubes and grown to an OD_600_ of 0.4. Next, 5 ml of bacterial culture was added to 10 ml of RNAprotect Bacteria reagent (Qiagen), vortexed for 10 s, and incubated at room temperature for 5 min. Next, all tubes were centrifuged at 4,400 × *g* for 15 min at room temperature, supernatants were discarded, and bacterial pellets were frozen at −80°C (the pellet was stored before use for no longer than 1 week).

RNA isolation was done with an RNeasy Mini spin kit (Qiagen). Next, RNA was digested with RNase-free DNase (Qiagen). RNA quantity and purity was checked with NanoDrop, and RNA quality was determined with an Agilent 2100 Bioanalyzer.

RNA was sequenced with the use of the HiSeq X platform at the Sanger Institute Sequencing Facility. The library for sequencing was prepared with a NEB Ultra II RNA custom kit (NEB). Sequencing quality was assessed on the basis of average base quality, GC content, and adapter contamination (49). Reads were mapped to the reference genomes of *S*. Typhimurium SL1344 and *S*. Enteritidis P125109, respectively, and counted with the use of the Rsubread package (64). Differential expression analysis was performed with the use of edgeR (65), and a gene was considered differentially expressed when the false discovery rate was equal to or lower than 0.05.

### Data processing and statistical analysis.

Statistical analysis was performed using R software (66). All figures were prepared with the ggplot2 package implemented in R software (67).

For screening of the 127 *Salmonella* isolates, results from three experiments (nine measurements in total) were baselined using results from empty-well controls. First, to eliminate the interreplicate variance, median infection value (number of bacteria per square millimeter) for each of three replicates from one measurement was computed. Next, the final infection value (number of bacteria per square millimeter) was computed as a median of medians from three experiments. To facilitate comparison between measurements from different plates, final median values were standardized using the median value of the SL1344 strain for a given plate. The procedure was repeated for three cell lines and both incubation periods. Standardized data were later compared using Mood test with Benjamini-Hochberg correction for multiple comparisons. Statistical analysis for growth curves and gentamicin protection assays was performed using Wilcoxon test implemented in R software.

### Data availability.

Genome sequence and RNA-seq data for Salmonella enterica have been deposited at the NCBI under the BioProject numbers PRJNA626643 and PRJNA667254, respectively.

## Supplementary Material

Supplemental file 1
