# Supplementary material for: Identification of Natural Mutations Responsible for Altered Infection Phenotypes of Salmonella enterica Clinical Isolates by Using Cell Line Infection Screens
Source: Appl Environ Microbiol. 2021 Jan 4;87(2):e02177-20. doi: 10.1128/AEM.02177-20 (PMC7783345; doi:10.1128/AEM.02177-20)
Supplement: Supplemental file 1 [file AEM.02177-20-s0001.pdf]

Supplementary Table 1.
List of reference genomes (GenBank accession numbers in brackets) for each serovar used in this study.

Serovar	Ref. genome
Enteritidis	P125109 (GCF_000009505_1)
Typhimurium	LT2 (GCF_000006945_2)<br>SL1344 (GCF_000210855_2)
Dublin	ATCC_39184 (GCF_001953035_1)
Choleraesuis	SC B67 (GCF_000008105_1)
Gallinarum	ATCC_9120 (GCF_000330485_2)

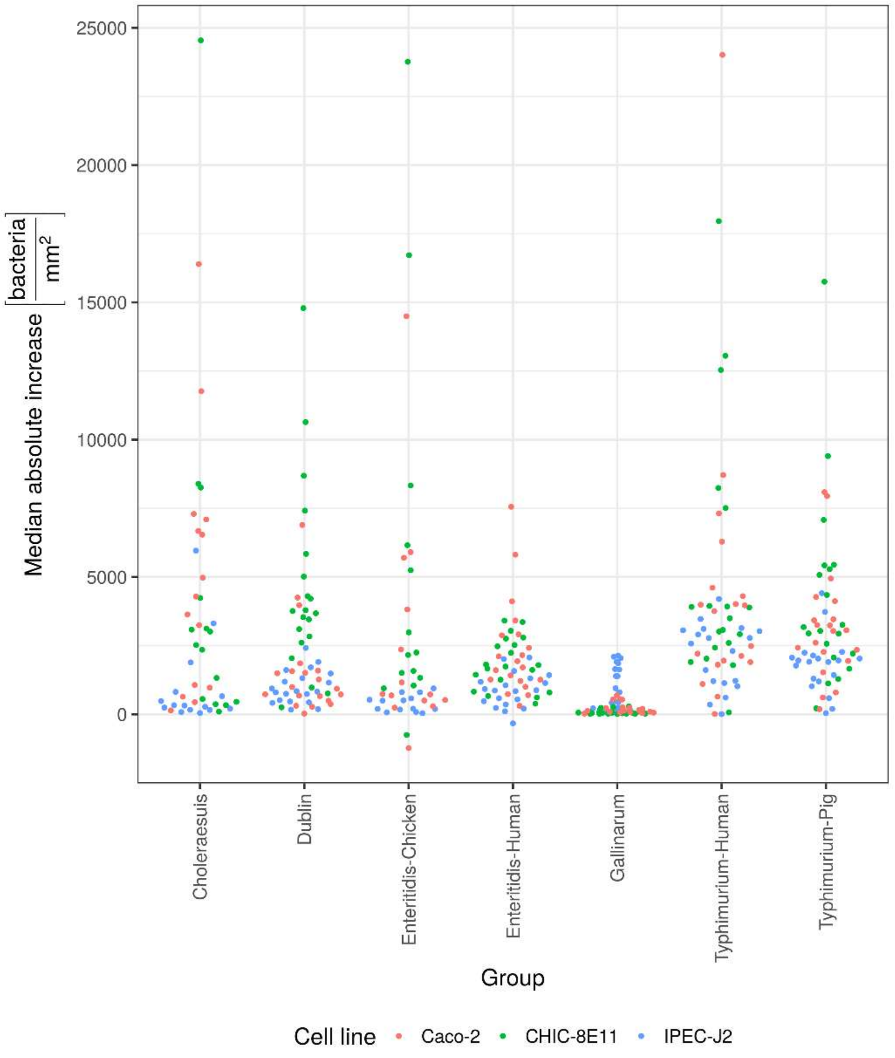

Supplementary Figure 1. Median absolute increase in infecting bacteria between incubation times

Salmonella isolates from different serovars and isolation sources were compared for the increase of bacteria between incubation times. Each dot represents an increase in infecting bacteria between two

incubation times ΔInf (ΔInf=Inf_{4h}-Inf_{1h}) for each isolate in one cell line (IPEC-J2- blue, Caco-2- red, CHIC-8E11- green). Results are shown as number of bacteria per mm^2.

Supplementary Table 2
Median infectivity [bacteria/mm^2] of *Salmonella* towards IPEC-J2, Caco-2 and CHIC-8E11 cells.

	1h	4h
IPEC-J2	52	1045
Caco-2	70	1955
CHIC-8E11	301	3154

Supplementary Table 3

Increase in infecting bacteria between two incubation times ΔInf (ΔInf=Inf_{4h}-Inf_{1h}, [bacteria/mm^2]) on IPEC-J2, Caco-2 and CHIC-8E11 cells for *Salmonella* isolates from *S*. Typhimurium of human (Typhimurium-Human) and pig (Typhimurium-Pig) origin, *S*. Enteritidis of human (Enteritidis-Human) and chicken (Enteritidis-Chicken) origin, *S*. Choleraesuis (Choleraesuis), *S*. Dublin (Dublin), *S*. Gallinarum (Gallinarum)

	IPEC-J2	Caco-2	CHIC-8E11
Typhimurium-human	2576	3759	3496
Typhimurium-pig	1936	3047	3107
Enteritidis-human	871	1820	1765
Enteritidis-chicken	350	730	2205
Choleraesuis	318	4289	2522
Dublin	833	962	3720
Gallinarum	947	128	47

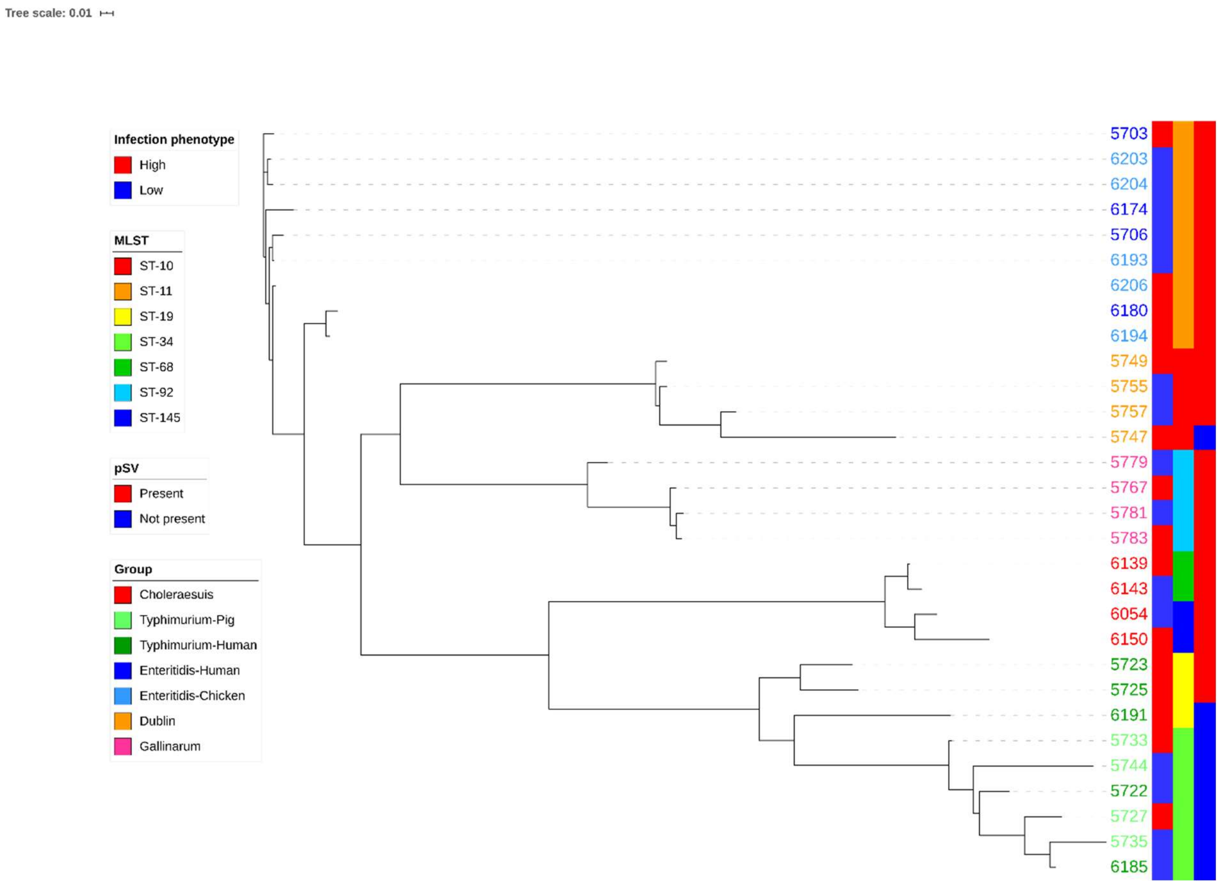

Supplementary Figure 2. Accessory genome binary tree of *Salmonella* with different infection phenotypes

Accessory genome binary tree was generated during annotated genomes were analysis with the Roary software. Information about infection phenotype, group, MLST sequence types and presence of pSV plasmid were added with use of iTOL. Groups and isolate numbers used in analysis: Choleraesuis-6054, 6139, 6143, 6150; Typhimurium-Pig- 5727, 5733, 5735, 5744; Typhimurium-Human-5722, 5723, 5725, 6185, 6191; Enteritidis-Human- 5703, 5706, 6174, 6180; Enteritidis-Chicken- 6193, 6194, 6203, 6204, 6206, Dublin- 5747, 5749, 5755, 5757; Gallinarum- 5767, 5779, 5781, 5783.

Supplementary Table 4. Growth of 5706 isogenic strains.
Data presented in table are medians (and median absolute deviations) from 3 independent experiments with 3 replicates for each strain.

Time of growth [h]	Factor	5706	5706_dksA_5706	5706_dksA_P125109	5706 vs 5706_dksA_P125109	5706_dksA_5706 vs 5706_dksA_P125109
10 (Exponential)	Growth rate (r)	0.68 (±0.01)	0.6 (±0.08)	1.16 (±0.03)	$p < 10^{-5}$	$p < 10^{-5}$
	Doubling time (t_gen)	1.01 (±0.02)	1.15 (±0.15)	0.6 (±0.01)	$p < 10^{-5}$	$p < 10^{-5}$
	AUC	0.75 (±0.24)	0.79 (±0.22)	1.69 (±0.11)	$p < 10^{-5}$	$p < 10^{-5}$
16 (Whole measurement time)	Growth rate (r)	0.97 (±0.02)	0.91 (±0.03)	1.03 (±0.02)	$p < 10^{-3}$	$p = 0.01$
	Doubling time (t_gen)	0.71 (±0.01)	0.77 (±0.02)	0.67 (±0.01)	$p < 10^{-3}$	$p = 0.01$
	AUC	3.96 (±0.27)	4.11 (±0.18)	5.06 (±0.23)	$p < 10^{-5}$	$p < 10^{-5}$

Abbreviations: AUC- area under curve

Supplementary Table 5. Number of differentially expressed genes for *S.* Typhimurium and *S.* Enteritidis isolates

Isolate number	5727	5735	6185	6191		Isolate number	5703	6174	6203	6206
5727	0	85	56	218		5703	0	15	47	67
5735	85	0	48	324		6174	15	0	64	44
6185	56	48	0	226		6203	47	64	0	1
6191	218	324	226	0		6206	67	44	1	0

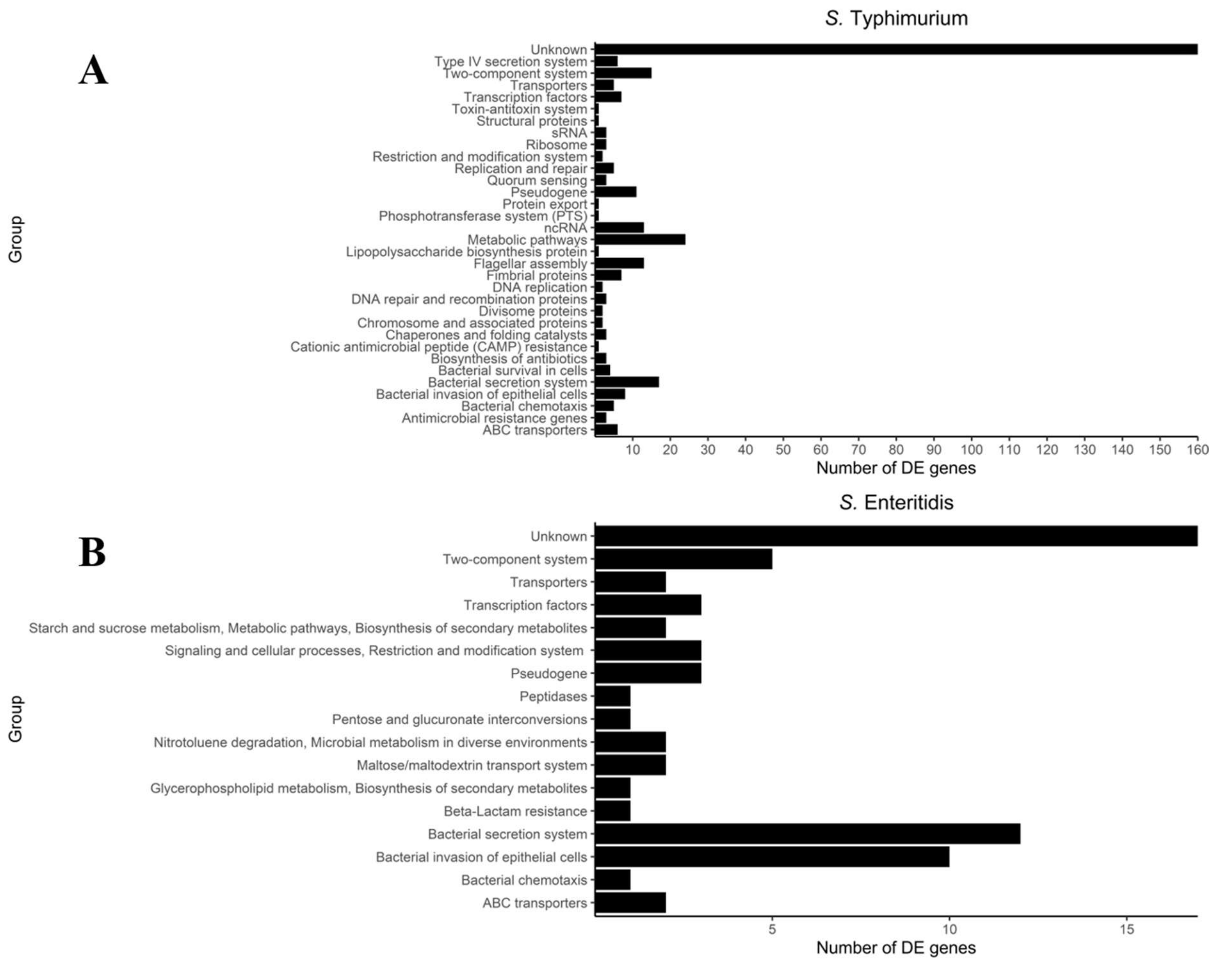

Supplementary Figure 3. Summary of functional classes of genes differentially expressed among *S.* Typhimurium (A) and *S.* Enteritidis (B) isolates with altered infection phenotypes.

Differentially expressed (DE) genes were grouped by their function (y-axis) and the number of DE genes for each group were summarized (y-axis).
